# How (not) to draw philosophical implications from the cognitive nature of concepts: the case of intentionality

**DOI:** 10.3389/fpsyg.2014.00799

**Published:** 2014-07-22

**Authors:** Kazuki Iijima, Koji Ota

**Affiliations:** ^1^Brain Science Institute, Tamagawa UniversityTokyo, Japan; ^2^Japan Society for the Promotion of ScienceTokyo, Japan; ^3^Department of Basic Science, Graduate School of Arts and Sciences, The University of TokyoTokyo, Japan

**Keywords:** experimental philosophy, normativism, descriptivism, Knobe effect, intentionality, theory of mind

## Abstract

Philosophers have often appealed to intuitive judgments in various thought experiments to support or reject particular theses. Experimental philosophy is an emerging discipline that examines the cognitive nature of such intuitive judgments. In this paper, we assess the methodological and epistemological status of experimental philosophy. We focus on the Knobe effect, in which our intuitive judgment of the intentionality of an action seems to depend on the perceived moral status of that action. The debate on the philosophical implications of the Knobe effect has been framed in terms of the distinction between the competence and performance of the concept of intentionality. Some scholars seem to suggest that the Knobe effect reflects the competence (or otherwise, the performance error) of the concept of intentionality. However, we argue that these notions are purely functional and thus do not have philosophical implications, without assuming normativism, which we see as problematic in a psychological methodology. Finally, focusing on the gap between competence and rationality, we suggest future directions for experimental philosophy.

## COMPETENCE AND PERFORMANCE IN EXPERIMENTAL PHILOSOPHY

Since the beginning of the 21st century, a new research program, “experimental philosophy,” which systematically studies the nature of intuition with psychological methodologies, has become increasingly popular (Knobe and Nichols, 2008a; Alexander, 2012; Knobe et al., 2012; Knobe and Nichols, 2013). In this paper, we consider why philosophical arguments are not informed by the identification of the cognitive natures of philosophical concepts. In what follows, while we mainly focus on the concept of intentionality, our arguments are generalizable to other concepts, such as that of free will.

In constructing or criticizing theories, philosophers have appealed to intuitive judgments in thought experiments, as seen in discussions of epistemology (Gettier, 1963), philosophy of mind (Searle, 1980; Chalmers, 1996), philosophy of language (Kripke, 1980), and so on. In doing so, philosophers have appealed to the fact that their intuitive judgments in thought experiments reflect their own theories. However, experimental philosophy has found that people’s intuitive judgments are affected by unexpected factors, beyond those conceived by philosophers.

One famous example is intuitive judgments on the intentionality of action (Knobe, 2003). Participants were presented with a story about a person working in a company. The story indicates that the person is working on a new business program and knows that its side effect will harm the environment. However, the person says that he does not care about this outcome and carries out the program, which leads to the bad side effect (“Harm” condition). Another version of the story is the same, except that the side effect of the program is good for the environment (“Help” condition). The participants read one of these stories and were asked whether the person has intentionally harmed (helped) the environment. Surprisingly, a strong asymmetry was found between the two conditions: most participants in the Harm condition responded that his action was intentional, while most in the Help condition replied that it was not intentional, despite the identical structure of the stories. Thus, people’s intuitive judgments on the intentionality of an action vary, according to the perceived harmfulness/helpfulness of the action. It seems that, generally speaking, we attribute intentionality to harmful or morally bad side effects. This tendency of attribution is called the “Knobe effect.”

The Knobe effect may reflect the concept of intentionality. Intentionality attribution, which is the crucial function of our “theory of mind” (ToM), is partly driven by moral cognition in nature and is thus the result of the appropriate application of the concept of intentionality. In this case, the concept of intentionality can be regarded as being constituted by moral cognition. However, the Knobe effect may reflect the inappropriate interference of moral cognition that is not constitutive of the concept of intentionality (Nadelhoffer, 2004, 2006). This question about the nature of the Knobe effect has often been framed in terms of *competence* and *performance*. The Knobe effect reflects the competence of the concept of intentionality (i.e., the core of ToM) or an error in the performance of it (Alexander, 2012).

The distinction between competence and performance originates in generative linguistics. Chomsky (1965, p. 3) argues that competence is linguistic knowledge that is possessed by an ideal speaker-listener in a language community. Because the actual linguistic performance is affected by a variety of constraints, such as memory capacities, attention controls, vocal functions, and so on, language competence is perfectly reflected in performance only in the idealization of these functions. This distinction has yielded noteworthy results by factoring out heterogeneous, confounding factors from the main target of linguistic theories (see discussion by Jackendoff, 2002).

There is an ongoing debate on how to characterize psychologically the relationships between competence and performance (Phillips, 2004; Marantz, 2005; Neeleman and van de Koot, 2010; Phillips and Lewis, 2013). Neeleman and van de Koot (2010) argue that competence and performance should be understood as theories of the same language system but at different descriptive levels. In this case, competence and performance would roughly correspond to different levels of analysis, i.e., the computational and algorithmic levels introduced by Marr (1982). However, we will not discuss this issue further in this paper, and we hereafter use the term competence/performance in Chomsky’s (1965) original sense, which seems to be dominant in the literature in experimental philosophy.

The distinction between competence and performance can be applied to concepts. The Knobe effect may reflect the competence of the concept of intentionality, such that moral cognition underlies the application of the concept. For example, Knobe (2006, p. 226) states that “moral considerations are playing a helpful role in people’s underlying competence itself.” Otherwise, the Knobe effect is a sort of performance error, where moral cognition distorts the application of the concept. Generally speaking, when a judgment involving a concept is affected by some psychological factor, it may reflect the competence of the concept, or it may be the result of error in its performance.

We can also pose this type of question in relation to many other studies. For instance, people’s intuitive judgments about free will have been examined, focusing on whether the concept of free will is compatible with determinism. Some scholars argue that the concept of free will is compatibilist, since the participants attribute free will to fictional characters in a deterministic world (Nahmias et al., 2005). However, other studies suggest that people’s judgments are sensitive to whether they are presented with abstract or concrete scenarios. People attribute free will in concrete scenarios much more than they do in abstract ones (Nichols and Knobe, 2007; cf. De Brigard et al., 2009; Mandelbaum and Ripley, 2012). Although this may be the case, an issue regarding the nature of the concept of free will remains to be resolved. Even if our attribution of free will is affected by the perception of concreteness, it is unclear whether such an effect reflects the competence of the concept of free will.

Interestingly, some scholars seem to suggest that the nature of people’s concepts has philosophical implications. When discussing how to interpret the Knobe effect, Adams and Steadman (2004, p. 173) mention the philosophical view that intentionality does not require intention, which the Knobe effect “may be taken to support.” In discussing free will, Nahmias et al. (2006, p. 30) maintain that “[b]ecause the free will debate is intimately connected to ordinary intuitions and beliefs via these values and practices, it is important that a philosophical theory of free will accounts for and accords with ordinary people’s understanding of the concept and their judgments about relevant cases.” In discussing the general background of experimental philosophy, Knobe and Nichols (2008b, p. 12) state, “[m]ore and more, philosophers are coming to feel that questions about how people ordinarily think have great philosophical significance in their own right.” Indeed, these scholars often seek to grasp the concepts of intentionality and free will in terms of competence/performance.

Here two questions arise. First, do experimental results, such as the Knobe effect, reflect the competence of the intentionality concept or a mere performance error? Second, how and why does such an understanding inform philosophical debates about intentionality? In what follows, we consider these two questions in turn.

## DEVELOPMENTAL AND DISABILITY STUDIES

Practices in linguistics may provide a clue in distinguishing between competence and performance. In linguistics, when competing theories possess identical explanatory powers, the possibility of language acquisition has been successfully used to constrain the range of theory (Chomsky, 1965; Yang, 2010). Moreover, agrammatism, which is a type of aphasia specific to syntactic processing, has been useful in clarifying domain-specific linguistic competence by dissociating domain-general components (Friedmann and Grodzinsky, 1997; Kinno et al., 2009). In a similar way, developmental and disability studies of the concept of intentionality may also help us to theorize the nature of the concept.

First, the cognitive nature of the concept of intentionality may be clarified by considering developmental studies of the Knobe effect. According to the experimental study of Leslie et al. (2006a), children as young as four showed the same tendency as adults. Moreover, the Knobe effect appeared as soon as the children learned to understand the concept of “do not care [bad side effects]” that was included in the experiment’s scenario. These results suggest that our innate concept of intentionality grows and fits with the Knobe effect. Segal (2008) argues, “it is difficult to believe that they learned it from observation of adult patterns of judgment, or that they inferred it from something else. It looks as though this is just how FP [folk psychology] grows” (ibid, p. 101). Thus, the Knobe effect is essentially associated with the concept of intentionality within ToM and reflects the competence of this concept.

Second, the cognitive nature of the concept of intentionality may be further clarified by considering people with autism spectrum disorder, who generally show some impairment in the ToM. On the autism spectrum, adults with Asperger’s syndrome or high-functioning autism (hereafter, AS/HFA) have no apparent disabilities in general intelligence and language and can generally pass a simple false-belief task, which is a simple test of the ToM. Moreover, with regard to basic moral perception, several studies have shown that there is no large difference between people on the autism spectrum and people with typical development (Blair, 1996; Grant et al., 2005; Leslie et al., 2006b). People with AS/HFA have difficulties, however, in understanding the mental states of others in complex situations and in passing higher level ToM tests, which involve sarcasm, irony, bravado, and the like. From these observations, it has been suggested that adults with AS/HFA use heuristics, which differ from the core of ToM, in order to understand the minds of others (Happé, 1995; Tager-Flusberg and Joseph, 2003). Thus, people with AS/HFA have impairments in the core of the ToM and use specific heuristics for the attribution of intentionality to complement these impairments, while their basic moral perceptions are normal.

A hypothesis regarding the Knobe effect in people with AS/HFA can be derived from these assumptions. If the Knobe effect reflects the competence of the concept of intentionality (i.e., the core of the ToM), we will not observe it in people with AS/HFA who have impairments in the core. Otherwise, if the Knobe effect is not a manifestation of the core of the ToM but is at best only a sign of heuristics in intentionality attribution, we will observe it in people with AS/HFA. In the light of the current empirical literature, the latter possibility is likely. A recent study of the Knobe effect in the autism spectrum group has revealed that even people with AS/HFA show the Knobe effect similarly to people with typical development (Zalla and Leboyer, 2011). Although further studies were needed, in this case, the Knobe effect would be regarded as a sign of heuristics in intentionality attribution and therefore irrelevant to the nature of the concept of intentionality.

As shown above, disability and developmental studies offer us a theoretical advance in understanding the nature of the Knobe effect. At the moment, there is conflicting evidence from such studies regarding whether the Knobe effect reflects competence.

## NORMATIVIST PSYCHOLOGY AND EXPERIMENTAL PHILOSOPHY

Here, we question the implications of the above findings. What are the philosophical implications of the fact that the Knobe effect reflects competence or performance error? One related philosophical problem is the relationship between intentionality and *intention*. While it seems that intention is required for an action to be intentional, some philosophers reject this conclusion (cf. Bratman, 1984; Adams, 1986). If the Knobe effect exactly reflects the competence of the intentionality concept, then the idea of disconnecting intentionality and intention may be supported, since intentionality could be attributed to the side effects of an action that were not intended (and such an attribution reflects the competence of the concept of intentionality). However, we would like to point out that an assumption is required for this philosophical implication.

The assumption is that the distinction between competence and performance error implies the distinction between the rationality and irrationality of concepts. Generally speaking, when a particular intuitive judgment cannot be regarded as rational, a philosophical argument based on this judgment is not justified, even if the intuitive judgment reflects the competence of the related concept. For example, when the Knobe effect does not reflect a rational thought, philosophical arguments on intentionality need not necessarily take the Knobe effect into account, even if the concept of intentionality is constituted by moral cognition. Here, we tentatively characterize the rationality of thought, including judgments and reasoning, as the disposition to produce true beliefs. This type of rationality, which has been regarded as essential in philosophical discussions, is called *epistemic* rationality, and it is distinct from other types of rationality, such as instrumental or ecological rationality. As long as our main concern is how we *should* think about the nature of intentionality, than we *do* think about it; there is no reason for philosophical theories to take into account whether the Knobe effect reflects competence, since it does not guarantee epistemic rationality.

Here, we can follow Elqayam and Evans (2011) in making a distinction between *descriptivism* and *normativism* in psychology. In general, normativist psychology directly relates competence and performance error to rational and irrational thought, respectively. By assuming particular norms, normativist psychology classifies thoughts as rational or irrational, depending on whether the nature of the thoughts accords with certain norms, and judges the distinction between the competence and performance error of the thoughts. Elqayam and Evans (2011) argue that psychology should follow linguistics, which adopts descriptivism and proposes a theory regarding the competence of language. In other words, psychology should dedicate itself to describe competence in accordance with descriptivism. They claim that psychology may distinguish competence and performance, but it should not engage in judging whether thoughts are rational or irrational. Otherwise, it draws *ought* from *is*, whose inference is generally unsupported.

In the same way, we want to point out that the distinction between competence and performance error has philosophical implications, only when we adopt normativism and assume the following norm: To be rational in the sense of being disposed to produce true beliefs, thoughts should reflect the competence of concepts. Indeed, if we assume this norm, we obtain the following consequence: If the Knobe effect reflects competence, it can be regarded as a *rational judgment* and thus should be considered in philosophical arguments. However, we suggest that how we *ought to* think about the nature of intentionality has to be distinguished from how typically developed people *do* think about it in everyday life.

Let us consider the above research on disabilities of the ToM where people with AS/HFA evince the Knobe effect. From this fact, we might infer that the Knobe effect reflects the heuristics but not the competence of the concept of intentionality (the core of the ToM). However, this view alone does not lead to the conclusion that the Knobe effect is not a rational judgment. In order to obtain such a conclusion, we must also assume that judgments in accordance with the functioning of the core can be regarded as rational. This assumption is a normative assertion regarding human thoughts. On what ground do thoughts based on the core have priority in terms of rationality? The fact that they were acquired in typical development cannot offer the reason, since the reason why typical development is directed to rational thought is questioned here. Whether the Knobe effect reflects competence is not directly relevant to truths about intentionality, since one cannot infer norms, at least in psychological facts, that make some judgments rational and others irrational.

Thus, we cannot draw philosophical implications without normativism, even while we can identify the cognitive concepts in terms of competence and performance. This requirement of normativism has not been explicated in the debates within experimental philosophy. While the importance of whether the Knobe effect reflects the competence of the concept of intentionality has often been suggested, the idea of competence/performance does not guarantee anything like (ir)rationality. Notice that this is also the case in linguistic studies, where the idea of competence is a kind of biological function, which implies nothing about epistemic rationality. This is true regardless of the general characterization of biological functions. Even if our moral cognition has some causal role in the application of the concept of intentionality or if the interaction between moral cognition and intentionality attribution is important for biological adaptation, it has nothing to do with how often we arrive at the truth about intentionality (cf. Stich, 1990). Therefore, we conclude that there is a large gap between the idea of competence, which is a kind of biological function, and (ir)rationality.

## CONCLUSIONS

We suggest that it is empirically possible to determine whether the application of the concept of intentionality reflects its competence or error in performance. However, we point out that this fact about competence/performance does not imply anything positive about the nature of intentionality, contrary to the assumption made by some scholars in experimental philosophy. Drawing these implications from competence/performance requires a normativist psychology, which we think is a doubtful methodology. Thus, we have to bring something from outside psychology to bridge the gap between the competence/performance and (ir)rationality of concepts. For example, we might suggest that the concept of intentionality works for social interaction (cf. Knobe, 2006) and thus that socially admitted norms about its use should be reflected in any theory of intentionality. Otherwise, we might suggest a constitutive approach in general, which claims that our intuitions produce conceptual truth “by drawing on constructs such as reflective equilibrium and constitutive norms” (Evans and Elqayam, 2011, p. 283; cf. Thomasson, 2012). While we do not believe that experimental results do not inform philosophy, it seems better to explore something beyond the competence/performance of concepts. In any case, we have to be aware of the gap between how we should think and how we think about intentionality.

## Conflict of Interest Statement

The authors declare that the research was conducted in the absence of any commercial or financial relationships that could be construed as a potential conflict of interest.
